# Upcycling Leather Waste Through Zero-Waste Hydrolysis for Versatile 3D Printable Composites

**DOI:** 10.3390/polym17172366

**Published:** 2025-08-30

**Authors:** Giovanni Venturelli, Luca Guida, Marinella Levi

**Affiliations:** Department of Chemistry, Materials and Chemical Engineering “Giulio Natta”, Politecnico di Milano, Piazza Leonardo da Vinci 32, 20133 Milan, Italy; marinella.levi@polimi.it

**Keywords:** leather waste, hydrolyzed leather, Direct Ink Writing, vat photopolymerization, circular economy

## Abstract

The leather industry produces a substantial amount of solid waste, which is frequently disposed of via incineration or landfilling. While hydrolysis offers a valuable and sustainable method to chemically recycle leather waste, both acidic and alkaline processes present challenges due to the salts produced during neutralization. This study aims to upcycle leather scraps through hydrolysis, producing a powdered filler for versatile composites suitable for both LCD vat photopolymerization and Direct Ink Writing 3D printing technologies. A zero-waste hydrolysis process was adopted using sulfuric acid neutralized with calcium hydroxide, achieving a yield of 91.3%. The composites featured a matrix composed of polyethylene-glycol-diacrylate and glycerol dimethacrylate, with embedded leather hydrolysate powder at concentrations up to 20% *w*/*w_matrix_*. Tensile tests conducted on neat resin and composites demonstrated the strengthening effect of leather hydrolysate filler. Additionally, rheological tests displayed a viscoelastic behavior suitable for the adopted 3D printing technologies. The composites were successfully 3D-printed using both Direct Ink Writing and vat photopolymerization techniques, showing promising printing accuracy. This work demonstrates the potential of valorizing leather waste, upcycled via a hydrolysis method, to produce composites suitable for additive manufacturing to advance the sustainability and the circularity of the fashion sector.

## 1. Introduction

The tanning and finishing processes of leather have significant environmental impacts due to the use of hazardous chemicals and the production of wastewater. Moreover, only 20% of the treated animal hides mass is effectively converted into marketable leather, generating large amounts of solid waste.

Currently, leather scraps are primarily disposed of through landfilling or incineration [1,2]. To mitigate the environmental impact, research has focused on recycling and valorizing tanneries’ solid waste as a sustainable alternative to conventional disposal methods, in line with the principles of the circular economy. In recent years, efforts have been made to recycle leather solid waste mechanically: leather shavings (LS) were employed as solid fillers [3,4] and flame retardants [5], and were embedded in natural rubber to produce leather alternatives [6]. However, the mechanical recycling of LS as solid fillers is limited by their fibrous structure, which constrains the amount of upcycled leather that can be incorporated as filler in circular composites. Additionally, the hygroscopicity of leather particulate complicates their processing, hindering effective pulverization [4].

Chemical recycling, particularly hydrolysis, offers a promising alternative to overcome the limitations of mechanical recycling. Hydrolysis enables the breakdown of the leather’s collagen network into lower molecular weight oligopeptides. The resulting dried hydrolysate is a brittle and glassy solid that can be finely pulverized and incorporated into polymeric matrices [7,8,9]. The most used methods for collagen extraction rely on its hydrolysis through acidic solutions, as well as in alkaline solutions [10]. However, a significant drawback of leather hydrolysis processes is the formation of soluble salts during the neutralization step. The removal of these byproducts is complex and time- and resource intensive, thus limiting process scalability [11].

In the context of circular and sustainable industrial processes, additive manufacturing (AM) is a material-efficient technology featuring lower material waste and energy consumption compared to traditional subtractive manufacturing techniques [12,13]. Direct ink writing (DIW) and LCD vat photopolymerization are AM technologies that do not require heating of the material and can be considered cold processing techniques [4].

DIW is an extrusion-based technology that has been successfully employed for the processing of diverse classes of pseudoplastic materials such as viscous pastes, hydrogels, inks, and cross-linkable resins [14,15,16]. Moreover, DIW facilitates the reprocessing of various virgin and recycled feedstocks, such as biomass-based pastes, ceramic scraps, and fiber-reinforced composites [17].

LCD-based vat photopolymerization is characterized by relatively good dimensional resolution and surface finish [18]. For this purpose, LCD 3D printing is used for microfluidic devices and medical models requiring feature accuracy less than 100 µm [19]. This process employs vector scanning and mask projection methods to solidify photopolymer resin at a specific wavelength. It has gained considerable attention from both academia and industry due to its rapid processing speed and diverse applications in fields such as functional devices, ceramics, and biomedical engineering [20].

Although there is growing interest in AM for processing recycled feedstocks, a significant amount of leather waste remains underutilized [21]. Moreover, despite the growing interest in circular materials, the upcycling of leather waste for 3D-printable composites remains largely underexplored. Only a limited number of studies have investigated the mechanical recycling of leather in the context of AM [3,4,22], and no prior work has exploited hydrolyzed leather for 3D printing applications. Indeed, one of the main obstacles in the adoption of hydrolysis processes is the formation of soluble salts during neutralization, which can hinder scalability and limit industrial applicability.

In this context, this study presents a sustainable strategy for recycling LS via acidic hydrolysis to produce a filler suitable for 3D printable composites. During the hydrolysis process, the adoption of sulfuric acid and calcium hydroxide resulted in the formation of calcium sulfate (gypsum) as a neutralization byproduct, which was easily separated from the hydrolysate. The pulverized leather hydrolysate was incorporated as a filler into a polymer matrix and 3D printed with DIW and LCD-based vat photopolymerization 3D printing technologies.

By coupling chemical recycling with AM, this work demonstrates the feasibility of integrating upcycled fillers and potentially bio-based resins [23] into mechanically tunable, environmentally sustainable, circular, and versatile 3D printable composites.

## 2. Materials and Methods

### 2.1. Leather Shavings Hydrolysis

LS were recovered from the market with a granulometry up to 4 mm. LS hydrolysis was conducted by mixing 1M sulfuric acid (H_2_SO_4,_ Sigma-Aldrich, St. Louis, MO, USA) at a 1:6.25 (*w*/*v*) ratio for 3 h at 90 °C under mechanical stirring. The resulting suspension was vacuum filtered to remove non-hydrolyzed residues.

Successively, the acidic leather hydrolysate was neutralized by gradual addition of calcium hydroxide (Ca(OH)_2_, BioKi, Collecorvino, Italy) under stirring. The neutralization led to the precipitation of calcium sulfate (CaSO_4_), according to the following reaction:

H_2_SO_4_ + Ca(OH)_2_ → CaSO_4_ + 2H_2_O (1)

Calcium sulfate (gypsum) was separated from the hydrolysate by vacuum filtration. The hydrolyzed LS (HLS) solution was then dried at 70 °C, ground into a fine powder using a jade mortar and pestle and employed as filler in composite formulations.

### 2.2. Composite Formulations

Composite formulations were prepared using a matrix composed of Polyethylene Glycol Diacrylate (PEGDA, Mw 700, Sigma-Aldrich, St. Louis, MO, USA) and Glycerol dimethacrylate (GDMA, Sigma-Aldrich, St. Louis, MO, USA) at a ratio of 80:20. Hereinafter, *w_matrix_* indicates the total mass of the two matrix monomers. Ethyl phenyl (2,4,6-trimethyl benzoyl)phosphinate (TPO-L, 3% *w*/*w_matrix_*, Lambson Limited, Wetherby, UK), and dicumyl peroxide (0.5% *w*/*w_matrix_*, Sigma-Aldrich, St. Louis, MO, USA) were added as photo initiator and thermal initiator, respectively.

For LCD vat photopolymerization, pulverized HLS was introduced as a filler at a concentration up to 20% *w*/*w_matrix_*. For DIW, HLS was added up to 20% *w*/*w_matrix_*, together with 4% *w*/*w_matrix_* fumed silica (Sigma-Aldrich, St. Louis, MO, USA) as a rheological modifier. Each formulation is named according to the intended technology and the percentage of hydrolysate included. For example, DIW10 refers to the formulation designed for DIW, containing 10% HLS filler and fumed silica.

### 2.3. Rheological Properties

Flow and strain sweep tests evaluated the rheological behavior of composite formulations with a rotational rheometer Discovery HR2 (TA instrument, New Castle, DE, USA). A plate-plate geometry with a diameter of 20 mm and a 400 μm gap was chosen. Rheological tests were performed at 25 °C.

During flow tests, the shear rate was logarithmically increased from 10^−1^ s^−1^ to 10^3^ s^−1^, recording three points per decade. In the strain sweep tests, an oscillatory strain was applied from 0.01% to 1000% with a frequency of 1 Hz.

### 2.4. DIW Printability Assessment

The slicing process of stl models produced with Fusion360 (version 2.0.19941) was carried out with the open-source slicing software PrusaSlicer (Prusa Research, Prague, Czech Republic, version 2.5.0). DIW 3D printing process was carried out using a custom-designed platform equipped with multiple extruders [24]. Extrusion was performed at 25 °C, setting a speed of 10 mm min^−1^, using an 18 G conical nozzle and 0.4 mm as layer height.

Printability tests were carried out to evaluate the shape fidelity of DIW10 and DIW20 by calculating three dimensionless parameters. Pores area, perimeters, and filament thickness were measured using Fiji software (version 2.14.0) in triplicates.

Specifically, the spreading factor (SF) was determined as the ratio of the printed filament diameter ($D_{F}$ [μm]) to the nozzle diameter ($D_{N}\;[\mu m]$):

(2) $$SF\;=\frac{D_{F}}{D_{N}}.$$

The measurements were performed on five filaments, with diameters recorded at seven random points along each filament.

Uniformity index (U) was calculated to evaluate the flow stability and homogeneity of the extruded material using the following equation:

(3) $$U=\frac{\sum_{i=1}^{n}\left|\bar{D}-D_{i}\right|}{\bar{D}}$$

where $\bar{D}[\mu m]$ is the average diameter of the printed filament, $D_{i}[\mu m]$ is the measured filament diameter, and *n* is the number of measurements performed. Three filaments were evaluated by measuring their diameters at seven random points.

Pore Index (Pr) was evaluated to quantify the material’s ability to reproduce corners and sharp edges accurately. A 30 × 30 mm^2^ square with a 70% grid infill pattern was 3D printed. The index is defined as [25]

(4) $$P_{r}=\frac{l^{2}}{16A},$$

where *l* [μm] and *A* [${\mu m}^{2}$] represent the perimeter and area of each pore, respectively. Seven pores were analyzed.

Additionally, a filament fusion test was performed by printing coil patterns with loops spaced at increasing distances. The ratio *f_s_*/*f_t_*, where *f_s_* is the fused filament length at the loop’s peak and *f_t_* is the filament thickness measured at five random points, was plotted against filament distance (*f_d_*) [26]. This test assessed the material’s capability to accurately replicate features such as closely spaced printed filaments and sharp curves.

### 2.5. LCD Printability Assessment

LCD vat photopolymerization was performed with Anycubic Photon Mono 4K LCD 3D printer (Anycubic Inc., Shenzhen, China). The slicing process of stl model produced with Fusion 360 (Autodesk) was carried out with Chitubox Pro V2.

Printability of LCD10 and LCD20 was optimized by minimizing the UV bottom exposure, defined as the UV curing time applied to the initial layers deposited directly onto the build platform to ensure sufficient polymerization and plate adhesion of the printed structure. Cubic specimens (5 × 5 × 2 mm^3^) were printed with bottom exposure times ranging from 5 to 40 s.

Subsequently, layer exposure, which is the UV curing time applied to all subsequent layers, was optimized. This parameter governs dimensional accuracy and interlayer bonding while preventing over-polymerization. Test structures with pillars and circular holes with diameters ranging from 0.5 mm to 2 mm were printed in triplicates to assess shape fidelity. The UV layer exposure ranged between 3 and 15 s. The pillar and hole diameters were measured with Fiji software in triplicates.

### 2.6. Mechanical Characterization

The mechanical properties of circular composites and pristine material (LCD0) were evaluated through uniaxial tensile tests following the ASTM standard test method D3039/D3039M-17 [27], using a Zwick Roell Z010 (ZwickRoell GmbH & Co. KG, Ulm, Germany) dynamometer equipped with a 10 kN load cell. Rectangular specimens with a length of 70 mm, a width of 10 mm, and a thickness of 2 mm were tested at a loading rate of 2 mm/min with a gauge length of 40 mm, without the use of extensometers. At least three specimens for every formulation were tested. Characterization of formulations for LCD printing (LCD0, LCD10, LCD20) were performed on 3D printed samples. Characterization of formulations for DIW (DIW10 and DIW20) was performed on samples produced by casting into silicone molds. All samples were cured for 1 h at 110 °C, followed by 1 h at 140 °C.

## 3. Results and Discussion

### 3.1. Leather Shavings Hydrolysis

**Figure 1:**
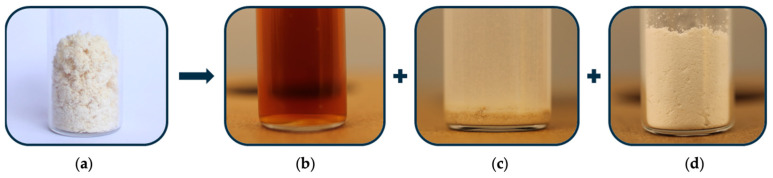
(**a**) Leather shavings used for hydrolysis. Products of hydrolysis: (**b**) liquid fraction, (**c**) solid residue, and (**d**) calcium sulfate.

Leather hydrolysis resulted in a solution and a solid fraction that was separated through an initial vacuum filtration (Figure 1). After neutralization and drying, the hydrolysate corresponded to 91.3% of the initial leather waste mass, demonstrating the high efficiency of the hydrolysis process.

The separation of soluble salts from the hydrolysate is typically challenging and may not be practical for the upcycling of industrial waste due to the production of large quantities of soluble salt (e.g., NaCl or Na_2_SO_4_). In the adopted hydrolysis process, the resulting neutralization salt is gypsum (CaSO_4_), insoluble in water, thus easily separable from the leather hydrolysate. Moreover, calcium sulfate is a valuable raw material, contributing to zero-waste and circular material production processes.

### 3.2. Rheological Characterization

Composite formulations for both LCD vat photopolymerization and DIW were tested to evaluate their rheological behavior and suitability for the two adopted 3D printing technologies.

In LCD vat photopolymerization 3D printing, excessively viscous resins can hinder the flow of uncured resin into the gap between the printed part and the UV light source during platform movements. This flow is essential to replace the uncured resin after each layer. If the resin is not replenished correctly, the same portion of resin may remain in place and be unintentionally cured. This can result in reduced printing accuracy and lead to the adhesion of the printings to the bottom of the vat [28].

**Figure 2:**
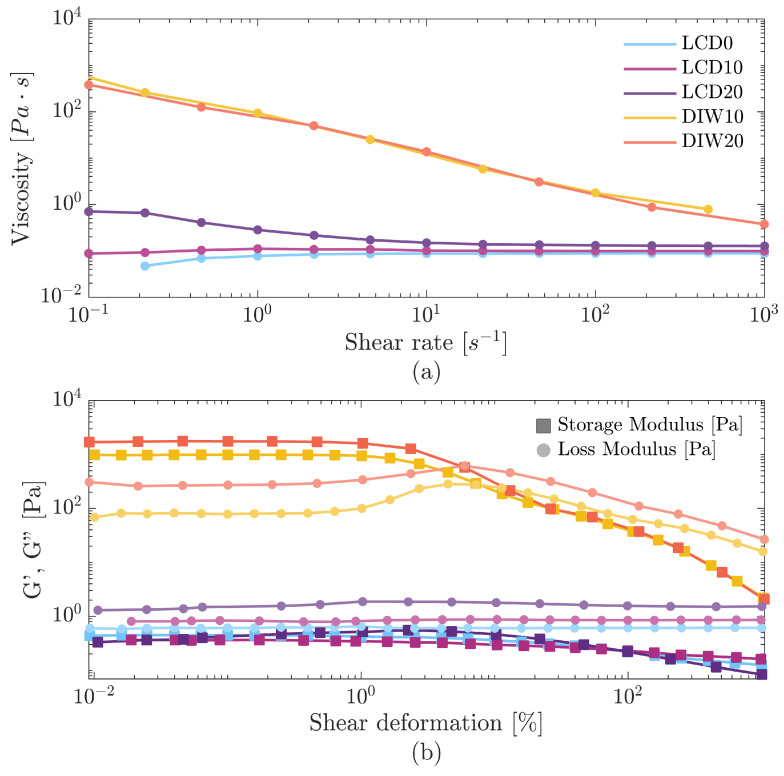
Rheological behavior of composite formulations: (**a**) flow curves and (**b**) strain sweep tests.

Filler addition increased ink viscosity: LCD10 and LCD20 exhibited higher viscosity value than LCD0 (Figure 2a). Moreover, for every LCD formulation, the strain sweep test showed no yield stress (Figure 2b), thus the materials did not exhibit any solid-like behavior maintaining continuous flow. Therefore, LCD formulations are suitable for LCD 3D printing but not for DIW.

DIW requires shear-thinning behavior and yield stress to ensure proper extrusion and shape retention. Shear thinning, observed as a decrease in viscosity with increasing shear rate, facilitates smooth flow through the nozzle during extrusion.

DIW10 and DIW20 exhibited decreasing viscosity with increasing shear rate (Figure 2a), indicating that composites with embedded fumed silica have a pseudo-plastic behavior, necessary for enabling a controlled material extrusion through the nozzle during the printing step. Amplitude sweep (Figure 2b) displayed a storage modulus (G′) higher than the loss modulus (G″) at small deformations, indicating solid-like behavior [29]. Such behavior is essential for shape fidelity, since it avoids the post-extrusion flow of material.

The yield point, defined as the crossover of G′ and G″, occurs at 7.23% and 5.95% strain for DIW10 and DIW20, respectively. The corresponding yield stress values are 29 Pa and 38 Pa.

The increase in G′ observed for both formulations can be attributed to interactions between silica and the resin matrix, and to the addition of the leather hydrolysate filler. Before the yield point, DIW formulations exhibited a higher G′ than LCD formulations, due to the presence of silica. Among them, DIW20 showed a slightly higher G′ than DIW10, consistent with its higher filler content. After the yield point, these interactions are disrupted, leading to a drop in G′ by approximately two orders of magnitude, and both formulations display similar behavior. Finally, DIW20 exhibited a slightly higher G″, likely due to greater energy dissipation associated with its increased filler content (Figure 2b).

The absence of yield stress in the LCD formulations and its presence in the DIW formulations confirmed that the addition of fumed silica as a rheological modifier was both beneficial and necessary. Its inclusion imparted the required yield stress to the DIW formulations, ensuring their suitability for the DIW process.

### 3.3. DIW Printability Assessment

**Figure 3:**
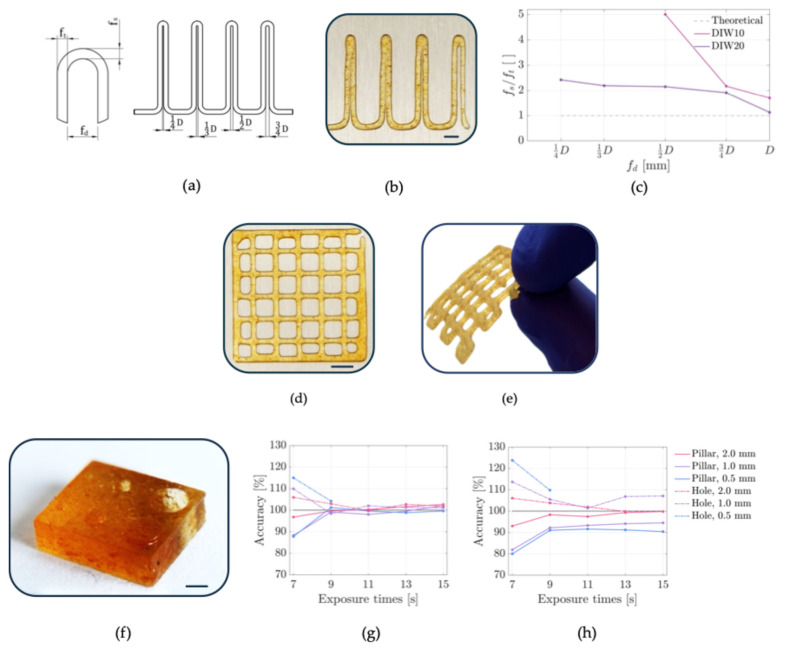
(**a**) Parameterized coil for filament fusion evaluation; (**b**) three-dimensionally printed coil with DIW (scale bar: 5 mm); (**c**) ratio between fused filament length and filament thickness (*f_s_*/*f_t_*) against filament distance (*f_d_*) for filament fusion assessment; (**d**) three-dimensionally printed grid with DIW (scale bar: 5 mm); (**e**) UV-cured 3D printed grid; (**f**) three-dimensionally printed sample through vat photopolymerization for printing accuracy evaluation (scale bar: 2 mm); (**g**) printing accuracy of LCD10 and (**h**) LCD20.

Filaments 3D printed through DIW displayed a spreading factor (SF, Table 1), a dimensionless parameter dependent on printing settings, that closely matched the ideal unitary value. Consequently, the measured filament diameters were consistent with the employed nozzle size (0.80 mm). The 3D-printed composites also showed a uniformity index (U, Table 1) near the ideal value, indicating the smoothness and homogeneity of the printed filaments. Additionally, the pore coefficient (Pr, Table 1), which assesses the pore aspect-ratio of 3D-printed grids (Figure 3d,e), was comparable to the unitary ideal value. These results highlight the materials’ capability to retain its shape after extrusion, enabling the printing of squared pores (Figure 3d). To further evaluate filament fusion, a coil with increasing loop distances (Figure 3a) was 3D-printed [26]. The plotted ratio (Figure 3c) demonstrated that as the filament distance (*f_d_*, Figure 3a) increased, the ratio (*f_s_*/*f_t_*) approached the unitary ideal value, resulting in a more uniform filament (Figure 3b). DIW20 demonstrated superior performance when compared to DIW10. This result is consistent with its higher yield stress measured during the amplitude sweep test (Figure 2b). The successful printings using DIW10 and DIW20 (Figure 3b,d,e) indicate their capability to accurately reproduce fine features, confirming their suitability for DIW applications.

### 3.4. LCD Printability Assessment

The optimization of the bottom layers’ UV exposure displayed a printing plate adhesion from 15 s of UV curing. Therefore, samples for shape fidelity evaluation were 3D printed with 15 s of UV bottom exposure to minimize the printing time while ensuring proper adhesion of constructs to the printing platform.

The evaluation of printing accuracy of composition suitable for LCD 3D printing (LCD10 and LCD20) was conducted on structures with pillars and holes (Figure 3f) printed at different times of UV exposure, to minimize printing time and simultaneously ensure shape fidelity. UV layer exposures of 3 and 5 s were not sufficient to cure the resin in either LCD10 or LCD20 formulations, leading to failed prints. In contrast, UV exposures between 7 and 15 s allowed monomer polymerization and yielded successful printing of pillars and holes. In both LCD10 and LCD20, extended UV exposure times improved dimensional accuracy, aligning the prints closely with the theoretical dimensions (Figure 3g,h). For both LCD10 and LCD20 formulations, 0.5 mm holes were open only at 7 and 9 s of UV layer exposure. LCD10 printings reached theoretical dimensions from 9 s of UV curing, while for LCD20, shape fidelity was satisfied only for 2 mm-structures cured for 13 and 15 s.

### 3.5. Mechanical Characterization

Pristine matrix (LCD0) and composites for 3D printing applications (LCD10, LCD20, DIW10, and DIW20) were tested to evaluate their mechanical properties, revealing the significant influence of the filler on the composite performance.

**Figure 4:**
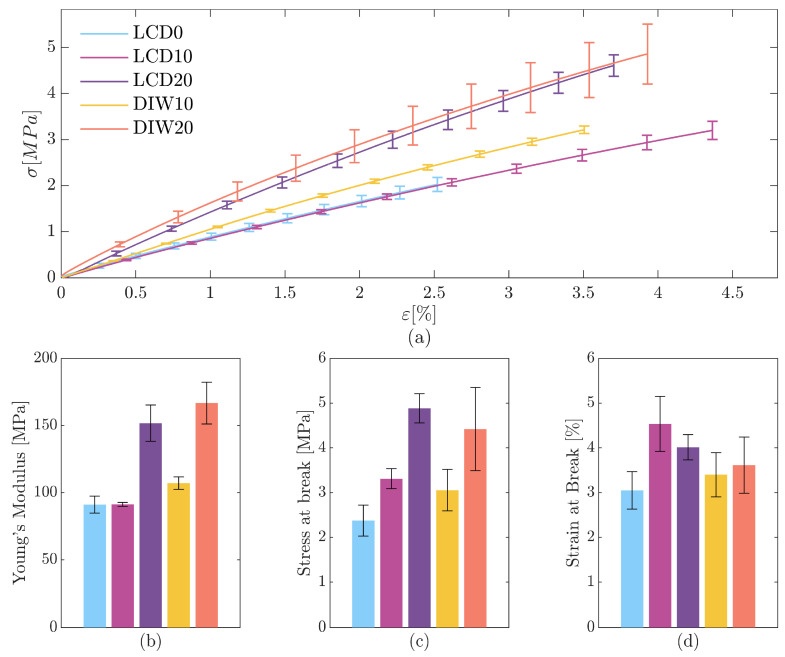
Mechanical characterization of composite formulations. (**a**) Stress–strain curves; (**b**) values of Young’s modulus; (**c**) values of stress at break; (**d**) values of strain at break.

The stress–strain curves of LCD0 and LCD10 (Figure 4a) exhibited similar behavior at small deformations, a result supported by the equivalent Young’s modulus reported in Figure 4b. This indicates that the addition of hydrolyzed leather filler to LCD0 does not significantly influence the stiffness of LCD10. In contrast, the higher resistance of LCD10 at larger strains can be attributed to interactions between the filler and the matrix, which enhance the material’s strength under higher deformation. In LCD20 formulation, the reinforcing effect of the circular filler is evident, with Young’s modulus increased to around 150 MPa and stress at break increased with respect to LCD10.

A slight increase in Young’s modulus is observed for the DIW10 composite, where fumed silica was incorporated as a rheological modifier. The stiffening and reinforcing effect of leather hydrolysate is evident when comparing DIW10 to DIW20 (Figure 4b), where both Young’s modulus and tensile strength increased with filler content.

When comparing composites with the same filler content (i.e., LCD10 and DIW10), LCD10 shows a slightly higher stress at break. However, the difference is not statistically significant, while a notably higher strain at break was observed. These improvements can be attributed to the superior surface finishing provided by vat photopolymerization 3D printing, which minimizes surface defects and thus reduces premature failures. A similar trend is observed at 20% filler content (LCD20 and DIW20), where both composites showed comparable stress at break. In addition, DIW samples displayed a larger standard deviation, possibly reflecting a higher defect density. The LCD20 sample demonstrated superior strain at break, proving that the better surface finish of LCD-printed specimens enhanced mechanical resistance under tensile loading. The use of recycled leather hydrolysate represents a sustainable strategy that enhances mechanical performance while enabling circular material utilization across two distinct 3D printing technologies.

Leather exhibits a modulus of 100 MPa, a strain at break of 10%, and a stress at break of 12 MPa [28], making the proposed composites not far from leather performances. By adjusting the ratio of GDMA, the rigid component, to PEGDA, the highly flexible component, it would be possible to match better the mechanical properties of leather. This suggests the suitability of the composite for applications in leather repair and customization, serving as a sustainable alternative material for use in the fashion industry.

## 4. Conclusions

The large amount of leather solid waste has spurred the optimization of processes and techniques to valorize leather scraps. In this work, acidic hydrolysis carried out under mild conditions has proven to be a promising strategy for valorizing leather scraps as filler for resin-based 3D-printable composites. The selection of the acid-base combination for hydrolysis and neutralization processes resulted in the formation of calcium sulfate. This byproduct can be easily removed and utilized as a raw material, ensuring a zero-waste process. Rheological tests showed that the composites exhibited viscoelastic properties suitable for DIW and vat photopolymerization 3D printing techniques. Indeed, printability assessments on structures printed with DIW and LCD vat photopolymerization displayed shape fidelity and retention. Moreover, the addition of leather hydrolysate as a filler in the PEGDA and GDMA matrix resulted in enhanced mechanical strength, with further enhancements expected by reducing the particle size of the filler. Ball milling offers a viable and practical method for achieving this finer particle size, which could enhance the dispersion and interaction of the filler within the matrix, leading to improved composite performances. This work demonstrates the potential of leather shavings, hydrolyzed through a sustainable protocol, to serve as filler in composites suitable for various additive manufacturing techniques, enabling the production of fashion-related accessories and the repair or both microscopic and macroscopic leather damage.

## Figures and Tables

**Table 1:** DIW printability parameters of composite formulations tested.

Composite	SF [-]	U [-]	P_r_ [-]
DIW10	0.87 ± 0.06	0.30 ± 0.04	0.88 ± 0.01
DIW20	0.90 ± 0.05	0.41 ± 0.09	0.87 ± 0.04

## Data Availability

The raw data supporting the conclusions of this article will be made available by the authors on request.
